# Supplementary material for: Dealing with aflatoxin B1 dihydrodiol acute effects: Impact of aflatoxin B1-aldehyde reductase enzyme activity in poultry species tolerant to AFB1 toxic effects
Source: PLoS One. 2020 Jun 22;15(6):e0235061. doi: 10.1371/journal.pone.0235061 (PMC7307737; doi:10.1371/journal.pone.0235061)
Supplement: S1 Table — (DOCX) [file pone.0235061.s001.docx]

| Feed ingredients (%) | Chicken diet | Turkey diet | Duck diet | Quail diet |
| --- | --- | --- | --- | --- |
| Corn | 55.0 | 49.1 | 51.5 | 47.5 |
| Corn gluten meal | --- | 4.5 | 5.0 | 3.5 |
| Wheat bran | --- | --- | 18.0 | --- |
| Full-fat soybean (extruded) | 11.7 | 6.0 | 2.0 | 6.8 |
| Soybean meal (48%) | 30.0 | 34.9 | 20.5 | 37.5 |
| Vegetable oil | 0.1 | 2.8 | --- | 1.0 |
| Calcium carbonate | 0.92 | 1.00 | 1.25 | 1.40 |
| Calcium phosphate (20% P) | 1.48 | 1.00 | 1.30 | 1.75 |
| Iodized salt | 0.3 | 0.2 | 0.2 | 0.35 |
| Vitamin:mineral premix | 1.0 | 1.0 | 1.0 | 1.0 |
| Methionine | 0.32 | 0.12 | 0.05 | 0.15 |
| Lysine | 0.10 | 0.25 | 0.12 | --- |
| Threonine | 0.03 | --- | --- | --- |
|  |  |  |  |  |
| Calculated analysis (%) | | | | |
| Crude protein | 25.1 | 25.5 | 20.1 | 26.1 |
| ME (kcal/kg) | 3103 | 3120 | 2800 | 2909 |
| Ether extract | 5.13 | 3.95 | 3.61 | 4.07 |
| Crude fiber | 2.77 | 3.24 | 4.08 | 3.33 |
| Linoleic acid | 1.25 | 1.49 | 1.42 | 1.29 |
| α-Linolenic acid | 0.22 | 0.15 | 0.08 | 0.16 |
| Calcium | 0.95 | 0.88 | 0.99 | 1.24 |
| Total phosphorus | 0.62 | 0.54 | 0.66 | 0.69 |
| Available phosphorus | 0.30 | 0.25 | 0.33 | 0.34 |
| Digestible lysine | 0.74 | 1.41 | 0.96 | 1.50 |
| Digestible methionine | 1.36 | 0.48 | 0.38 | 0.49 |
| Total sulphur amino acids | 0.48 | 0.83 | 0.72 | 0.85 |

**S1 Table**. Feed ingredients and nutritional content of the diets fed to the experimental birds.
