# Supplementary material for: Dealing with aflatoxin B1 dihydrodiol acute effects: Impact of aflatoxin B1-aldehyde reductase enzyme activity in poultry species tolerant to AFB1 toxic effects
Source: PLoS One. 2020 Jun 22;15(6):e0235061. doi: 10.1371/journal.pone.0235061 (PMC7307737; doi:10.1371/journal.pone.0235061)
Supplement: S2 Table — (DOCX) [file pone.0235061.s002.docx]

| Species | Sex | Body weight (g) | Feed Intake (kg/bird) | Species | Sex | Body weight (g) | Feed Intake (kg/bird) |
| --- | --- | --- | --- | --- | --- | --- | --- |
| Rhode Island Red chicks | Female | 750 | 1,07 | Quail | Female | 89,1 | 0,84 |
|  |  | 720 | 1,19 |  |  | 80,8 | 0,86 |
|  |  | 625 | 0,99 |  |  | 79,2 | 0,86 |
|  |  | 590 | 1,16 |  |  | 80,5 | 0,81 |
|  |  | 630 | 1,18 |  |  | 83,0 | 0,85 |
|  |  | 630 | 1,26 |  |  | 83,6 | 0,81 |
|  | Male | 865 | 1,29 |  | Male | 92,9 | 0,81 |
|  |  | 850 | 1,41 |  |  | 86,8 | 0,79 |
|  |  | 830 | 1,37 |  |  | 96,1 | 0,82 |
|  |  | 750 | 1,43 |  |  | 78,5 | 0,78 |
|  |  | 810 | 1,27 |  |  | 73,6 | 0,81 |
|  |  | 790 | 1,26 |  |  | 73,4 | 0,85 |
| Ross chicks | Female | 1600 | 4,25 | Turkey | Female | 2613 | 4,15 |
|  |  | 2850 | 4,15 |  |  | 2674 | 4,25 |
|  |  | 3175 | 4,23 |  |  | 3167 | 4,28 |
|  |  | 2175 | 4,48 |  |  | 2600 | 4,26 |
|  |  | 2450 | 4,15 |  |  | 2817 | 4,22 |
|  |  | 3200 | 3,81 |  |  | 2627 | 4,26 |
|  | Male | 3180 | 4,61 |  | Male | 3269 | 3,37 |
|  |  | 2900 | 4,55 |  |  | 2912 | 3,28 |
|  |  | 2750 | 4,78 |  |  | 2892 | 3,65 |
|  |  | 2850 | 4,50 |  |  | 3036 | 3,56 |
|  |  | 2550 | 4,78 |  |  | 2740 | 3,02 |
|  |  | 2760 | 4,53 |  |  | 3197 | 3,44 |
| Duck | Female | 1850 | 8,82 |  |  |  |  |
|  |  | 2640 | 8,69 |  |  |  |  |
|  |  | 2170 | 8,89 |  |  |  |  |
|  |  | 2300 | 8,92 |  |  |  |  |
|  |  | 2000 | 8,64 |  |  |  |  |
|  |  | 2100 | 8,70 |  |  |  |  |
|  | Male | 2700 | 7,77 |  |  |  |  |
|  |  | 2500 | 7,65 |  |  |  |  |
|  |  | 3100 | 7,79 |  |  |  |  |
|  |  | 2800 | 7,68 |  |  |  |  |
|  |  | 2600 | 7,95 |  |  |  |  |
|  |  | 2350 | 7,71 |  |  |  |  |

**S2 Table**. Final body weight and total feed intake at the time of sacrifice of the experimental birds.
