# Supplementary material for: Dealing with aflatoxin B1 dihydrodiol acute effects: Impact of aflatoxin B1-aldehyde reductase enzyme activity in poultry species tolerant to AFB1 toxic effects
Source: PLoS One. 2020 Jun 22;15(6):e0235061. doi: 10.1371/journal.pone.0235061 (PMC7307737; doi:10.1371/journal.pone.0235061)
Supplement: S3 Table — Values in bold represent individuals which ratio value is below SD. (DOCX) [file pone.0235061.s003.docx]

| **Species** | **Sex** | **Bird** | **dhd/dial** | **dhd/mono** | **dial/mono** | **mono/dial** |
| --- | --- | --- | --- | --- | --- | --- |
| ross | female | 1 | 2.73 | 2.72 | 1.00 | **1.00** |
| ross | female | 2 | 1.09 | **0.74** | 0.67 | 1.48 |
| ross | female | 3 | 1.74 | 1.57 | 0.90 | 1.11 |
| ross | female | 4 | 1.40 | **0.64** | **0.46** | 2.20 |
| ross | female | 5 | 2.88 | 1.29 | **0.45** | 2.23 |
| ross | female | 6 | 3.99 | 2.31 | 0.58 | 1.73 |
| ross | male | 1 | 1.27 | 0.94 | 0.74 | 1.36 |
| ross | male | 2 | 1.21 | 1.16 | 0.96 | 1.04 |
| ross | male | 3 | 7.27 | 2.13 | **0.29** | 3.41 |
| ross | male | 4 | 4.73 | 2.70 | 0.57 | 1.75 |
| ross | male | 5 | 1.69 | 1.04 | 0.62 | 1.62 |
| ross | male | 6 | 3.25 | 2.66 | 0.82 | 1.22 |
| island | female | 1 | 11.14 | 5.02 | 0.45 | 2.22 |
| island | female | 2 | 11.13 | 3.49 | **0.31** | 3.19 |
| island | female | 3 | 7.97 | **3.06** | **0.38** | 2.61 |
| island | female | 4 | 5.74 | 4.04 | 0.70 | 1.42 |
| island | female | 5 | 6.11 | 4.53 | 0.74 | 1.35 |
| island | female | 6 | 6.16 | 3.73 | 0.61 | 1.65 |
| island | male | 1 | 5.25 | 3.29 | 0.63 | 1.60 |
| island | male | 2 | **3.53** | 3.84 | 1.09 | **0.92** |
| island | male | 3 | 6.83 | 4.00 | 0.59 | 1.71 |
| island | male | 4 | 10.08 | 4.23 | 0.42 | 2.38 |
| island | male | 5 | 7.45 | **3.15** | 0.42 | 2.37 |
| island | male | 6 | 7.93 | 5.76 | 0.73 | 1.38 |
| quail | female | 1 | 18.22 | 18.44 | 1.01 | 0.99 |
| quail | female | 2 | **6.73** | 6.22 | 0.92 | 1.08 |
| quail | female | 3 | 9.67 | 9.28 | 0.96 | 1.04 |
| quail | female | 4 | 8.18 | 6.94 | 0.85 | 1.18 |
| quail | female | 5 | 29.19 | 17.06 | 0.58 | 1.71 |
| quail | female | 6 | **6.34** | **3.76** | 0.59 | 1.68 |
| quail | male | 1 | 40.84 | 29.43 | 0.72 | 1.39 |
| quail | male | 2 | 17.77 | 12.99 | 0.73 | 1.37 |
| quail | male | 3 | 14.70 | 7.25 | **0.49** | 2.03 |
| quail | male | 4 | 34.83 | 10.65 | **0.31** | 3.27 |
| quail | male | 5 | 19.59 | 9.87 | 0.50 | 1.98 |
| quail | male | 6 | 33.03 | 24.02 | 0.73 | 1.38 |
| turkey | female | 1 | 3.58 | 3.92 | 1.09 | **0.92** |
| turkey | female | 2 | 9.48 | 7.64 | 0.81 | 1.24 |
| turkey | female | 3 | 2.59 | 3.08 | 1.19 | **0.84** |
| turkey | female | 4 | 3.63 | 2.85 | 0.78 | 1.27 |
| turkey | female | 5 | 3.15 | 3.90 | 1.24 | **0.81** |
| turkey | female | 6 | 3.05 | 3.38 | 1.11 | **0.90** |
| turkey | male | 1 | 3.95 | 2.33 | **0.59** | 1.69 |
| turkey | male | 2 | 3.63 | 2.36 | **0.65** | 1.54 |
| turkey | male | 3 | 2.69 | **1.67** | **0.62** | 1.61 |
| turkey | male | 4 | 2.83 | 2.31 | 0.82 | 1.22 |
| turkey | male | 5 | 5.32 | 3.82 | 0.72 | 1.39 |
| turkey | male | 6 | 2.81 | 2.35 | 0.84 | 1.20 |
| duck | female | 1 | 208.75 | 81.43 | 0.39 | 2.56 |
| duck | female | 2 | 228.83 | 59.49 | **0.26** | 3.85 |
| duck | female | 3 | 361.73 | 84.35 | **0.23** | 4.29 |
| duck | female | 4 | 177.11 | 85.12 | 0.48 | 2.08 |
| duck | female | 5 | **44.56** | **23.13** | 0.52 | 1.93 |
| duck | female | 6 | 101.50 | 82.99 | 0.82 | 1.22 |
| duck | male | 1 | 181.93 | 182.60 | 1.00 | 1.00 |
| duck | male | 2 | **60.95** | 87.24 | 1.43 | **0.70** |
| duck | male | 3 | 102.01 | 139.39 | 1.37 | **0.73** |
| duck | male | 4 | **58.63** | **38.46** | 0.66 | 1.52 |
| duck | male | 5 | 142.59 | 81.06 | 0.57 | 1.76 |
| duck | male | 6 | 345.03 | 245.13 | 0.71 | 1.41 |

**S3 Table**. Individual values of CL_int_ AFB_1_-dhd enzyme production / CL_int_ AFB_1_ dialcohol enzyme production (dhd/dial), CL_int_ AFB_1_-dhd enzyme production / CL_int_ AFB_1_ monoalcohol enzyme production (dhd/mono), CL_int_ AFB_1_ monoalcohol enzyme production / CL_int_ AFB_1_ dialcohol enzyme production (mono/dial) and the inverse (dial/mono) ratios. Values in bold show the individuals with a ratio value below SD.
