# Supplementary material for: Dealing with aflatoxin B1 dihydrodiol acute effects: Impact of aflatoxin B1-aldehyde reductase enzyme activity in poultry species tolerant to AFB1 toxic effects
Source: PLoS One. 2020 Jun 22;15(6):e0235061. doi: 10.1371/journal.pone.0235061 (PMC7307737; doi:10.1371/journal.pone.0235061)
Supplement: Attachment — Submitted filename: Response to reviewers.docx [file pone.0235061.s004.docx]

**Point by point response to reviewers’ comments**

**Additional Editor Comments (if provided):**

The study is very well presented; I feel that the manuscript is dealing with a good topic but lacks in the quality of preparation. The main problem found in the manuscript is related to the methodology, other routes of detoxification were not discussed and typo errors or ambiguous phrases or sentences. It is necessary to improve the manuscript by examining the questions that need to be clarified in a way. Please be aware of the manuscript should be presented according to guidelines for authors of Plos One. For your guidance, you can check the reviewers' comments.

**Reply**: We would like to thank the editor for the time spent reviewing this manuscript. We have revised the document in order to improve its grammar, correct the typos and rephrase sentences that were not clear. Further, we have discussed other AFB_1_ biotransformation reactions that determine the ultimate toxic effect of the toxin. We hope that the revised document is now acceptable for publication in Plos One. Our itemized response to the raised queries follows below.

In regard to the manuscript adjustment to the Standards for Reporting Enzymology Data (STRENDA), the following changes have been done:

- K_M_ has been replaced by K_m_ everywhere in the manuscript.
- The EC number (1.1.1.2) of aflatoxin B_1_ aldehyde reductase enzyme has been added (line 13).
- The subcellular localization of the enzyme has been indicated (line 13).
- The reactions **reduction of AFB_1_ dialdehyde by AFAR: AFB_1_ dialdehyde + NADH + H^+^ → AFB_1_ monoalcohol + NAD^+^**; and **reduction of AFB_1_ monoalcohol by AFAR: AFB_1_ monoalcohol + NADH + H^+^ → AFB_1_ dialcohol + NAD^+^** have been added (lines 132 - 134).
- The reference by Judah *et al.* [8] has been added (reference for method assay) (line 134).
- The words “discontinuous direct assay” have been added in lines 137–138.
- V_max_ units were adjusted for AFB_1_ monoalcohol production. Instead of “nmol AFB_1_ dialdehyde/mg protein/minute”, the units are now presented as “nmol AFB_1_ monoalcohol/mg protein/minute” (lines 190,194–196).
- In order to be more specific, the K_m_ units have been rewritten from “µM” to “µM of AFB_1_ dialdehyde” (lines 198).

**Comments to the Author**

1. Is the manuscript technically sound, and do the data support the conclusions?

The manuscript must describe a technically sound piece of scientific research with data that supports the conclusions. Experiments must have been conducted rigorously, with appropriate controls, replication, and sample sizes. The conclusions must be drawn appropriately based on the data presented.

Reviewer #1: Yes

Reviewer #2: Yes

2. Has the statistical analysis been performed appropriately and rigorously?

Reviewer #1: Yes

Reviewer #2: I Don't Know

3. Have the authors made all data underlying the findings in their manuscript fully available?

The [PLOS Data policy](http://www.plosone.org/static/policies.action#sharing) requires authors to make all data underlying the findings described in their manuscript fully available without restriction, with rare exception (please refer to the Data Availability Statement in the manuscript PDF file). The data should be provided as part of the manuscript or its supporting information, or deposited to a public repository. For example, in addition to summary statistics, the data points behind means, medians and variance measures should be available. If there are restrictions on publicly sharing data—e.g. participant privacy or use of data from a third party—those must be specified.

Reviewer #1: Yes

Reviewer #2: Yes

 4. Is the manuscript presented in an intelligible fashion and written in standard English?

PLOS ONE does not copyedit accepted manuscripts, so the language in submitted articles must be clear, correct, and unambiguous. Any typographical or grammatical errors should be corrected at revision, so please note any specific errors here.

Reviewer #1: Yes

Reviewer #2: Yes

5. Review Comments to the Author

Please use the space provided to explain your answers to the questions above. You may also include additional comments for the author, including concerns about dual publication, research ethics, or publication ethics. (Please upload your review as an attachment if it exceeds 20,000 characters)

**Reviewer #1:**

This manuscript provides an evidence that differences in the resistance of poultry species to aflatoxin B1 toxicity are associated with differences in AFAR activity.

Please consider the following suggestions:

Klein et al (Comp Biochem Physiol C Toxicol Pharmacol. 2002 Jun; 132 (2): 193-201.) show that AFAR activity increases with age.
In this study, chickens were 7 weeks old, turkeys and quails were 8 weeks old, and Peking Duck was 9 weeks old. Please explain whether these age differences affect AFAR activity.

**Reply**: We are in complete agreement with the reviewer about the changes on the expression levels of AFAR enzyme associated with the bird’s age, as it was reported by Klein *et al.* (2002). Unfortunately, the study of Klein *et al*. (2002) does not present western immunoblot densitometry values to compare between ages; however, their data suggest that AFAR expression reaches a maximum level in turkeys at 41 days of age. Further, other two key enzymes involved in xenobiotic biotransformation (cytochrome P450s and glutathione sulfotransferases) also seem to reach a maximum enzyme activity at 41 days of age in turkeys.

Since the turkeys used in our study were 8 weeks old (56 day-old), they were expected to have reached their maximum AFAR activity. To our knowledge there are no data regarding the relationship between age and AFAR activity in the other poultry species studied. A sentence has been inserted indicating the fact that AFAR expression and activity change with age and that consideration must be given to this fact when analyzing the results of the present trial (lines 297 - 300).

Different poultry species may have different feeds.
Please explain the potential effects of different feed types, compositions, and feeding patterns on bioenzymatic activity.

**Reply**: We agree with the reviewer in that different types of feed may affect the patterns of bioenzymatic activity. However, all diets were formulated with the same macro-ingredients (corn, extruded full-fat soybeans, soybean meal, vegetable oil, calcium phosphate, calcium carbonate, etc.), adjusted for the specific requirements of each poultry species. Therefore, we do not expect an effect of the diet on the results obtained.

Ethoxyquin is an ingredient found in commercial feeds. It should be explained whether the diet used in this study contained ethoxyquin.

**Reply**: No ethoxyquin was added to the diets provided to the experimental birds.

L307-309: The present study... in chickens and ducks. ...in different poultry species?

**Reply**: The words “in chickens and ducks” have been replaced by “in different poultry species” (lines 335 - 336).

**Reviewer #2: GENERAL COMMENTS**

The MS provides a description of a study with potentially very important results. However, before the suggestions of selecting ducks with high AFAR activity, a larger variety of birds may need to be assessed to validate more clearly the population effect across a wider diversity of bird sources. Is there any evidence already that some ducks may be dramatically less susceptible to AF exposure than others?
The limitation of the study should be included in the discussion.

**Reply**: We agree with the reviewer in that the population of ducks used in our study is very limited. However, we chose the white Pekin duck in our study, which it is the type of duck most commonly used in commercial duck meat and egg production. We have added a sentence addressing this issue in the revised manuscript (lines 347 - 353).

It is not clear why other routes of detoxification were not discussed and this should be addressed. For example, enzyme activity in other organs may add to that of the activity in the liver (eg GST activity in the kidney).

**Reply**: We completely agree with the reviewer in that there are other routes of detoxication that are also important in AF metabolism. However, due to the fact that aflatoxin exposure is through the gastrointestinal tract and because all of the absorbed compounds from the GIT must pass first through the liver (because of the portal circulation), it is the liver the organ that is most important in AF biotransformation and detoxication. GST detoxication of the electrophilic metabolite of AFB_1_ (the 8,9-exo-epoxide, AFBO) is a key detoxication pathway that is also being investigated by our research group. What we want to point out in the present study is that tolerant poultry species like the chicken have a higher AFAR enzyme activity than sensitive poultry species like the duck. We do not intend to ignore the possible role of other metabolic pathways in AFB_1_ sensitivity. We only want to postulate that AFAR activity is probably contributing to the higher resistance of chickens to the adverse effects of AFB_1_.

**SPECIFIC COMMENTS**

TITLE
Change tolerance to tolerant: “… in poultry species TOLERANT to AFB1 toxic effects”

**Reply**: “Tolerance” has been replaced by “tolerant” in the title.

ABSTRACT
I suggest that the abstract would invite greater interest from potential readers if it stated more clearly WHAT IS KNOWN and WHAT IS ADDED BY THIS STUDY. It is an important and interesting subject, but one that is familiar to a very limited audience, an audience that could be increased by acknowledging that they are not abreast of the AFB1 literature.

**Reply**: This is a very important suggestion and we would like to thank the reviewer for it. In order to create a greater interest in the article the following sentence has been added at the beginning of the abstract: “Aflatoxin B_1_ aldehyde reductase (AFAR) enzyme activity has been associated to a higher resistance to the aflatoxin B_1_ (AFB_1_) toxicity in ethoxyquin-fed rats. However, no studies about AFAR activity and its relationship with tolerance to AFB_1_ have been conducted in poultry. To determine the role of AFAR in poultry tolerance, the hepatic *in vitro* enzymatic activity of AFAR was investigated in liver cytosol from four commercial poultry species (chicken, quail, turkey and duck)”

As captured later, please consider and respond to the following points:
Intrinsic clearance: from a pharmacological perspective, clearance relates to a particular organ. In the biochemical context that is described in the MS it applies only to the selected enzyme. This should be made very clear, as hepatic clearance may be far higher than a single enzyme clearance if more than one enzyme is involved.

**Reply**: We are in complete agreement with the reviewer’s comments regarding CL_int_.

We have inserted a sentence explaining that the calculated intrinsic clearance only applies for the selected enzymatic activity (not for the liver) and that due to the fact that we did not purify AFAR enzyme from hepatic cytosolic extracts, the calculated CL_int_ values are only “apparent” [Apparent Intrinsic Clearance (CL_intapp_)] (lines 128 – 129, 167 – 170).

Highest AF dialdehyde concentration tested (66mcM): how does this concentration relate to the expected exposure concentration in intoxicated birds?

**Reply**: The AFB_1_ concentration expected to occur in the hepatocyte upon AFB_1_ exposure is in the nanomolar or even the femtomolar order (Ch’in, J. & Devlin, T. The distribution and intracellular translocation of aflatoxin B_1_ in isolated hepatocytes. Biochem. Biophys. Res. Commun. 122, 1–8). In chickens dosed with a single intra-crop bolus of 2 mg of AFB_1_ per kg of body weight (a very large amount compared to what it is expected from a contaminated diet) AFB_1_ plasma concentrations have been only 96 nM (Lauwers, M., Croubels, S., De Baere, S., Sevastiyanova, M. 2019. Supplementary materials: Assessment of dried blood spots for multi-mycotoxin biomarker analysis in pigs and broiler chickens. Toxins 11: S1 – S8). Therefore, it is likely that under natural conditions AFB_1_ dialdehyde concentrations are not going to reach the 66 µM concentration, because the cytosolic AFB_1_ concentration is expected to be in the nanomolar range. We used high AFB_1_ dialdehyde concentrations in our *in vitro* model because of the need to saturate the enzymes; also we needed to be able to detect the analytes (the limit of quantitation of the analytical technique is 0.4 ng/g for AFB_1_).

The same AFAR enzyme catalyzes mono- and di-alcohol AFB1: is this the same enzyme or the same or related or linked enzyme?

**Reply**: The search conducted in databases like the NCBI or Kegg pathways indicates that the poultry AKR7A2 enzyme is responsible for the reduction of AFB_1_ dialdehyde and AFB_1_ monoalcohol. Because we found a strong relationship between these two enzyme activities (high Spearman’s rank-order correlation coefficient), we postulated that both activities are carried out by the same enzyme.

AFAR activity related to toxic effects in chickens and ducks – not quail and turkey: does not this imply that the relationship is complex and potentially multifactorial in all species?

**Reply**: We completely agree with the reviewer. AFAR activity is expected to depend on other reactions like glutathione sulfotransferase (GST) enzyme activity. In separate studies conducted by our research group we have found significant differences in GST activity among the same poultry species studied. Differences in the enzymatic nucleophilic trapping of AFBO will affect the production of AFB_1_ dihydrodiol and therefore the concentration of AFB_1_ dialdehyde. The overall metabolism of AFB_1_ most likely depends on several enzymatic reactions. A sentence has been added to clarify the multifactorial nature of the AFB_1_ detoxication pathways (lines 282 - 289).

INTRODUCTION
No comments (all good)

MATERIALS AND METHODS
Lines 100-104
Details of the breed/source of the turkeys, quail, and Pekin ducks should be provided, especially to allow comparison with any further studies of in vitro or in vivo activity of AFB1.

**Reply**: Details of the poultry used in the study have been provide as follows: Nicholas turkeys, Japanese quail (*Coturnix coturnix japonica*) (there are no Bobwhite quail -*Colinus virginianus*- in Colombia) and meat-type Pekin ducks (there are both meat-type and egg-type Pekin ducks (lines 102 - 104).

In addition, the composition of the diet of the birds prior to sacrifice should be described, especially the presence of any medications in the diet. For example, it would be common practice to include anticoccidial agents in the diet of chickens and special anticoccidial agents can cause changes in hepatic enzyme activity. Similarly, the main nutritional ingredients in the diet should be identified.

**Reply**: No additives or medication were added to the diets provided to the birds. The diets were formulated with the same ingredients (corn, extruded full-fat soybeans, soybean meal, vegetable oil, calcium phosphate, calcium carbonate, sodium chloride, lysine, methionine, tryptophan, choline, vitamin and mineral premix) formulated to reach or exceed the nutrient requirements of each poultry species studied. The ingredients and lack of use of non-nutritive additives have been indicated in the revised manuscript (lines 104 - 109).

Line 128
Is there a normal avian body temperature? Measurement of adult/mature bird body temperature has found a temperature of 41 for chickens, 41 for turkeys and 42 for ducks. Your comments on choice of a single temperature for all species and justification for the selection of 39 would be welcome. Younger birds may have a temperature closer to 39.

**Reply**: We agree with reviewer comment. Because the birds used in this trial were 7–9 weeks old birds, the expected temperature was around 39 degrees Celsius. The revised manuscript has been added with the words “the normal body temperature for the age of the birds used” (line 138 - 139).

Lines 150-160
The statistical significance level should be presented and the actual value in each test presented later in the MS

**Reply**: The following sentence has been added to the “Statistical analysis” section: “with a significance level of 5% (p<0.05).” (line 173). The actual p-value obtained in each test has been presented in the “Results” section where appropriate.

Line 155
Intrinsic clearance – the units should be provided. In addition, as each species has a different mass of liver tissue, the total liver clearance for the enzyme of interest is a useful parameter.

**Reply**: The CL_int_ units have been indicated: “mL/mg protein/minute” (lines 166-167). We agree with the reviewer comment about the concept of total liver clearance; however, since we did not purify the hepatic AFAR enzyme, we are not able to calculate the total liver clearance. We used the CL_int_ value to express the enzyme efficiency in the same way that it has been reported in previous studies [Guengerich, F. P. Analysis and characterization of enzymes and nucleic acids relevant to toxicology. In Hayes’s Principles and Methods of Toxicology Sixth Edition (eds Hayes, A. W. & Kruger, C. L.) 1939, ISBN-13: 978-1842145364 (CRC Press, 2014)]. Further, as it was mentioned previously, the CL_int_ value obtained corresponds to the apparent CL_int_.

RESULTS
Fig 2 and Fig 3 Captions
Why is the difference in MW of monoalcohol and dialcohol 2 units and not 1 unit?

**Reply**: AFB_1_ monoalcohol has an aldehyde function (R – CHO) that is reduced to alcohol, thus producing AFB_1_ dialcohol. In this carbonyl function, there is a double bond between carbon and oxygen (R – CH = O). When the carbonyl function is reduced, this C = O double bond becomes R – CH – O. One hydrogen is received by oxygen to produce the alcohol function (R – CH – OH) and the carbon atom receives the other hydrogen to complete its 4 bonds (R – CH_2_ – OH). That is why the molecular weight difference between the monoalcohol and the dialcohol is 2 units.

DISCUSSION
Line 248
… its capacity to FORM adduct …

**Reply**: The word “form” has been inserted as it was suggested (line 265)

Line 264
The fact that there is an apparent relationship between AFAR activity and AFB1 susceptibility only in ducks and chickens, and not in quail and turkeys, suggests that toxicity may be multifactorial. Some discussion of this would add increased credibility to the study.

**Reply**: We completely agree with the reviewer. The following paragraph has been added to the Discussion section: “It is important to highlight that AFAR enzyme activity cannot be considered as the only reaction capable of explaining the differences in sensitivity among different poultry species. For example, glutathione sulfotransferase (GST) enzyme activity is capable of affecting the production of AFB_1_ dihydrodiol (and therefore the production of AFB_1_ dialdehyde) through AFBO nucleophilic trapping. Therefore, the toxicity of AFB_1_ should be considered as a multifactorial mechanism in which different metabolic pathways in AFB_1_ biotransformation are interconnected, including AFAR activity” (lines 282 - 289).

Line 273-274
Does the correlation described indicate that the same enzyme is involved or suggest that the enzyme activities are related or linked? Why is the only conclusion that they are the same enzyme?

**Reply**: A linear correlation between two enzyme activities has been used in different experimental models to assume that a single enzyme is responsible for both enzyme activities (Eaton, D.L., Ramsdell, H.S., Neal, G. E. 1994. Biotransformation of aflatoxins. In: The toxicology of aflatoxins. Human health, veterinary, and agricultural significance. Eds. Eaton, D.L. and Groopman, J.D. Academic Press. 53 – 55; Shou, M., Dai, R., Cui, D., Korzekwa, K.R., Baillie, T.A., Rushmore, T.H. 2001. A kinetic model for the metabolic interaction of two substrates at the active site of cytochrome P450 3A4. J. Biol. Chem. 276: 2256 – 2262). Because of the high correlation between AFB_1_ dialdehyde reduction and AFB_1_ monoaldehyde reduction, we have hypothesized that the AKR7A2 member is likely responsible for both activities.

Line 276
How representative of the concentration present in the liver of intoxicated birds is the highest concentration of AFBI-dialdehyde of 66mcM?

**Reply**: As it was explained previously, the 66 µM concentration of AFB_1_ dialdehyde was used due to the conditions required by the experimental model (although the expected values are in the nanomolar range, not the micromolar range). However, because reduction products are normalized to the amount of cytosolic protein used, the kinetic parameters obtained are expected to reflect what actually happens in the hepatocyte.

Line 298
Is the cytotoxic effect of AFB1 exposure in ducks DUE TO or ASSOCIATED WITH the lack of a detoxification pathway? What is the status of GST detoxification pathways in ducks?

**Reply**: Currently there is no information on the enzyme kinetic parameters of GST activity against AFB_1_ or its metabolite AFBO in ducks. Kinetic parameters for this enzyme activity have been determined by our research group and these results are currently under consideration for publication. These unpublished studies have shown that the duck GST activity against AFBO is so low that the cytotoxic effects of AFB_1_ are most likely due to the lack of an appropriate detoxication pathway.

CONCLUSION
Line 317
The mention of individuals with high activity may relate to the SD values in Table 1. It would be useful to provide the individual bird values from which the Table was compiled – in this way the variation by individual birds and the number of birds with significant variation can be seen – this could be included in a supplement.

**Reply**: We agree with the reviewer regarding the identification of possible “high AFAR activity” individuals. Individuals with a dhd/dial or dhd/mono ratio value below average minus SD reflect individuals with the highest AFB_1_ dialdehyde reduction or AFB_1_ monoalcohol reduction efficiency (inactivation reactions) relative to the AFB_1_-dhd efficiency (activation reaction). The following chart has been added as supplementary material:

| **Species** | **Sex** | **Individual** | **dhd/dial** | **dhd/mono** | **dial/mono** | **mono/dial** |
| --- | --- | --- | --- | --- | --- | --- |
| ross | female | 1 | 2.73 | 2.72 | 1.00 | **1.00** |
| ross | female | 2 | 1.09 | **0.74** | 0.67 | 1.48 |
| ross | female | 3 | 1.74 | 1.57 | 0.90 | 1.11 |
| ross | female | 4 | 1.40 | **0.64** | **0.46** | 2.20 |
| ross | female | 5 | 2.88 | 1.29 | **0.45** | 2.23 |
| ross | female | 6 | 3.99 | 2.31 | 0.58 | 1.73 |
| ross | male | 1 | 1.27 | 0.94 | 0.74 | 1.36 |
| ross | male | 2 | 1.21 | 1.16 | 0.96 | 1.04 |
| ross | male | 3 | 7.27 | 2.13 | **0.29** | 3.41 |
| ross | male | 4 | 4.73 | 2.70 | 0.57 | 1.75 |
| ross | male | 5 | 1.69 | 1.04 | 0.62 | 1.62 |
| ross | male | 6 | 3.25 | 2.66 | 0.82 | 1.22 |
| island | female | 1 | 11.14 | 5.02 | 0.45 | 2.22 |
| island | female | 2 | 11.13 | 3.49 | **0.31** | 3.19 |
| island | female | 3 | 7.97 | **3.06** | **0.38** | 2.61 |
| island | female | 4 | 5.74 | 4.04 | 0.70 | 1.42 |
| island | female | 5 | 6.11 | 4.53 | 0.74 | 1.35 |
| island | female | 6 | 6.16 | 3.73 | 0.61 | 1.65 |
| island | male | 1 | 5.25 | 3.29 | 0.63 | 1.60 |
| island | male | 2 | **3.53** | 3.84 | 1.09 | **0.92** |
| island | male | 3 | 6.83 | 4.00 | 0.59 | 1.71 |
| island | male | 4 | 10.08 | 4.23 | 0.42 | 2.38 |
| island | male | 5 | 7.45 | **3.15** | 0.42 | 2.37 |
| island | male | 6 | 7.93 | 5.76 | 0.73 | 1.38 |
| quail | female | 1 | 18.22 | 18.44 | 1.01 | 0.99 |
| quail | female | 2 | **6.73** | 6.22 | 0.92 | 1.08 |
| quail | female | 3 | 9.67 | 9.28 | 0.96 | 1.04 |
| quail | female | 4 | 8.18 | 6.94 | 0.85 | 1.18 |
| quail | female | 5 | 29.19 | 17.06 | 0.58 | 1.71 |
| quail | female | 6 | **6.34** | **3.76** | 0.59 | 1.68 |
| quail | male | 1 | 40.84 | 29.43 | 0.72 | 1.39 |
| quail | male | 2 | 17.77 | 12.99 | 0.73 | 1.37 |
| quail | male | 3 | 14.70 | 7.25 | **0.49** | 2.03 |
| quail | male | 4 | 34.83 | 10.65 | **0.31** | 3.27 |
| quail | male | 5 | 19.59 | 9.87 | 0.50 | 1.98 |
| quail | male | 6 | 33.03 | 24.02 | 0.73 | 1.38 |
| turkey | female | 1 | 3.58 | 3.92 | 1.09 | **0.92** |
| turkey | female | 2 | 9.48 | 7.64 | 0.81 | 1.24 |
| turkey | female | 3 | 2.59 | 3.08 | 1.19 | **0.84** |
| turkey | female | 4 | 3.63 | 2.85 | 0.78 | 1.27 |
| turkey | female | 5 | 3.15 | 3.90 | 1.24 | **0.81** |
| turkey | female | 6 | 3.05 | 3.38 | 1.11 | **0.90** |
| turkey | male | 1 | 3.95 | 2.33 | **0.59** | 1.69 |
| turkey | male | 2 | 3.63 | 2.36 | **0.65** | 1.54 |
| turkey | male | 3 | 2.69 | **1.67** | **0.62** | 1.61 |
| turkey | male | 4 | 2.83 | 2.31 | 0.82 | 1.22 |
| turkey | male | 5 | 5.32 | 3.82 | 0.72 | 1.39 |
| turkey | male | 6 | 2.81 | 2.35 | 0.84 | 1.20 |
| duck | female | 1 | 208.75 | 81.43 | 0.39 | 2.56 |
| duck | female | 2 | 228.83 | 59.49 | **0.26** | 3.85 |
| duck | female | 3 | 361.73 | 84.35 | **0.23** | 4.29 |
| duck | female | 4 | 177.11 | 85.12 | 0.48 | 2.08 |
| duck | female | 5 | **44.56** | **23.13** | 0.52 | 1.93 |
| duck | female | 6 | 101.50 | 82.99 | 0.82 | 1.22 |
| duck | male | 1 | 181.93 | 182.60 | 1.00 | 1.00 |
| duck | male | 2 | **60.95** | 87.24 | 1.43 | **0.70** |
| duck | male | 3 | 102.01 | 139.39 | 1.37 | **0.73** |
| duck | male | 4 | **58.63** | **38.46** | 0.66 | 1.52 |
| duck | male | 5 | 142.59 | 81.06 | 0.57 | 1.76 |
| duck | male | 6 | 345.03 | 245.13 | 0.71 | 1.41 |

**S1 table**. Values of CL_int_ AFB_1_-dhd enzyme production / CL_int_ AFB_1_ dialcohol enzyme production (dhd/dial), CL_int_ AFB_1_-dhd enzyme production / CL_int_ AFB_1_ monoalcohol enzyme production (dhd/mono), CL_int_ AFB_1_ monoalcohol enzyme production / CL_int_ AFB_1_ dialcohol enzyme production (mono/dial) and the inverse (dial/mono) ratios. Values in bold represent individuals which ratio value is below SD.

Line 318-Line 319
Genetic selection would only be useful if there was a relationship between intoxication and enzyme activity. This would need to be established for the duck and may require challenge of ducks at the extremes (ie high and low) of enzyme activity with AF to see if in fact there is an in vitro in vivo (IVIV) relationship. Furthermore, only a very small sample of ducks from an unknown source has been studied and described herein.

**Reply**: We agree with the reviewer comment. We are fully aware of the limitations of the present study. Nonetheless we believe that we can postulate that research about genetic selection could potentially be used as a possible means of enhancing species tolerance. S1 Table gives additional support regarding the possibility of selecting individuals with a particularly high AFAR activity that could be selected for potentially higher tolerance to AFB_1_. As it has been mentioned in the first comment of reviewer #2, a sentence has been added to clarify the limitations of the present study in regard to the population studied (lines 347 - 353).
