# Supplementary material for: Dealing with aflatoxin B1 dihydrodiol acute effects: Impact of aflatoxin B1-aldehyde reductase enzyme activity in poultry species tolerant to AFB1 toxic effects
Source: PLoS One. 2020 Jun 22;15(6):e0235061. doi: 10.1371/journal.pone.0235061 (PMC7307737; doi:10.1371/journal.pone.0235061)
Supplement: Attachment — Submitted filename: Response to reviewers.docx [file pone.0235061.s005.docx]

**Point by point response to reviewers’ comments**

Reviewers' comments:

Reviewer's Responses to Questions

**Comments to the Author**

1. If the authors have adequately addressed your comments raised in a previous round of review and you feel that this manuscript is now acceptable for publication, you may indicate that here to bypass the “Comments to the Author” section, enter your conflict of interest statement in the “Confidential to Editor” section, and submit your "Accept" recommendation.

Reviewer #1: (No Response)

Reviewer #2: All comments have been addressed

2. Is the manuscript technically sound, and do the data support the conclusions?

The manuscript must describe a technically sound piece of scientific research with data that supports the conclusions. Experiments must have been conducted rigorously, with appropriate controls, replication, and sample sizes. The conclusions must be drawn appropriately based on the data presented.

Reviewer #1: Partly

Reviewer #2: Yes
3. Has the statistical analysis been performed appropriately and rigorously?

Reviewer #1: Yes

Reviewer #2: Yes
4. Have the authors made all data underlying the findings in their manuscript fully available?

The [PLOS Data policy](http://www.plosone.org/static/policies.action#sharing) requires authors to make all data underlying the findings described in their manuscript fully available without restriction, with rare exception (please refer to the Data Availability Statement in the manuscript PDF file). The data should be provided as part of the manuscript or its supporting information, or deposited to a public repository. For example, in addition to summary statistics, the data points behind means, medians and variance measures should be available. If there are restrictions on publicly sharing data—e.g. participant privacy or use of data from a third party—those must be specified.

Reviewer #1: Yes

Reviewer #2: Yes
5. Is the manuscript presented in an intelligible fashion and written in standard English?

PLOS ONE does not copyedit accepted manuscripts, so the language in submitted articles must be clear, correct, and unambiguous. Any typographical or grammatical errors should be corrected at revision, so please note any specific errors here.

Reviewer #1: Yes

Reviewer #2: Yes
6. Review Comments to the Author

Please use the space provided to explain your answers to the questions above. You may also include additional comments for the author, including concerns about dual publication, research ethics, or publication ethics. (Please upload your review as an attachment if it exceeds 20,000 characters)

**Reviewer #1**: Thank you for responding to my comments and taking appropriate opinions. However, in order to objectively understand the views expressed in this paper, there seems to be insufficient information on the experimental conditions as background. To the author's opinion that dietary effects on the results obtained are not expected, it is necessary to be objective by presenting as much evidence as possible of each poultry rearing status in this study. The authors should give supplemental data on the actual feed ingredients (mixing ratio and ME), feed intake, body weight, origin of rearing and notable clinical symptoms.

**Reply**: We want to thank again reviewer #1 for his constructive comments. In order to clarify rearing origin and poultry clinical signs (the word symptoms is usually restricted to human patients), the following phrase has been added to manuscript: “Poultry were obtained from local commercial poultry suppliers and at the moment of sacrifice no noticeable clinical signs were observed “ (lines 109 - 111).

In regard to the feed ingredient’s composition and calculated analysis (mixing ratio and ME), feed intake and body weight, tables S1 and S2 have been added as supplementary information. Former S1 table has been renumbered as S3 Table.

| Feed ingredients (%) | Chicken diet | Turkey diet | Duck diet | Quail diet |
| --- | --- | --- | --- | --- |
| Corn | 55.0 | 49.1 | 51.5 | 47.5 |
| Corn gluten meal | --- | 4.5 | 5.0 | 3.5 |
| Wheat bran | --- | --- | 18.0 | --- |
| Full-fat soybean (extruded) | 11.7 | 6.0 | 2.0 | 6.8 |
| Soybean meal (48%) | 30.0 | 34.9 | 20.5 | 37.5 |
| Vegetable oil | 0.1 | 2.8 | --- | 1.0 |
| Calcium carbonate | 0.92 | 1.00 | 1.25 | 1.40 |
| Calcium phosphate (20% P) | 1.48 | 1.00 | 1.30 | 1.75 |
| Iodized salt | 0.3 | 0.2 | 0.2 | 0.35 |
| Vitamin:mineral premix | 1.0 | 1.0 | 1.0 | 1.0 |
| Methionine | 0.32 | 0.12 | 0.05 | 0.15 |
| Lysine | 0.10 | 0.25 | 0.12 | --- |
| Threonine | 0.03 | --- | --- | --- |
|  |  |  |  |  |
| Calculated analysis (%) | | | | |
| Crude protein | 25.1 | 25.5 | 20.1 | 26.1 |
| ME (kcal/kg) | 3103 | 3120 | 2800 | 2909 |
| Ether extract | 5.13 | 3.95 | 3.61 | 4.07 |
| Crude fiber | 2.77 | 3.24 | 4.08 | 3.33 |
| Linoleic acid | 1.25 | 1.49 | 1.42 | 1.29 |
| α-Linolenic acid | 0.22 | 0.15 | 0.08 | 0.16 |
| Calcium | 0.95 | 0.88 | 0.99 | 1.24 |
| Total phosphorus | 0.62 | 0.54 | 0.66 | 0.69 |
| Available phosphorus | 0.30 | 0.25 | 0.33 | 0.34 |
| Digestible lysine | 0.74 | 1.41 | 0.96 | 1.50 |
| Digestible methionine | 1.36 | 0.48 | 0.38 | 0.49 |
| Total sulphur amino acids | 0.48 | 0.83 | 0.72 | 0.85 |

**S1 Table**. Feed ingredients and nutritional content of the diets fed to the experimental birds.

| Species | Sex | Body weight (g) | Feed Intake (kg/bird) | Species | Sex | Body weight (g) | Feed Intake (kg/bird) |
| --- | --- | --- | --- | --- | --- | --- | --- |
| Rhode Island Red chicks | Female | 750 | 1,07 | Quail | Female | 89,1 | 0,84 |
|  |  | 720 | 1,19 |  |  | 80,8 | 0,86 |
|  |  | 625 | 0,99 |  |  | 79,2 | 0,86 |
|  |  | 590 | 1,16 |  |  | 80,5 | 0,81 |
|  |  | 630 | 1,18 |  |  | 83,0 | 0,85 |
|  |  | 630 | 1,26 |  |  | 83,6 | 0,81 |
|  | Male | 865 | 1,29 |  | Male | 92,9 | 0,81 |
|  |  | 850 | 1,41 |  |  | 86,8 | 0,79 |
|  |  | 830 | 1,37 |  |  | 96,1 | 0,82 |
|  |  | 750 | 1,43 |  |  | 78,5 | 0,78 |
|  |  | 810 | 1,27 |  |  | 73,6 | 0,81 |
|  |  | 790 | 1,26 |  |  | 73,4 | 0,85 |
| Ross chicks | Female | 1600 | 4,25 | Turkey | Female | 2613 | 4,15 |
|  |  | 2850 | 4,15 |  |  | 2674 | 4,25 |
|  |  | 3175 | 4,23 |  |  | 3167 | 4,28 |
|  |  | 2175 | 4,48 |  |  | 2600 | 4,26 |
|  |  | 2450 | 4,15 |  |  | 2817 | 4,22 |
|  |  | 3200 | 3,81 |  |  | 2627 | 4,26 |
|  | Male | 3180 | 4,61 |  | Male | 3269 | 3,37 |
|  |  | 2900 | 4,55 |  |  | 2912 | 3,28 |
|  |  | 2750 | 4,78 |  |  | 2892 | 3,65 |
|  |  | 2850 | 4,50 |  |  | 3036 | 3,56 |
|  |  | 2550 | 4,78 |  |  | 2740 | 3,02 |
|  |  | 2760 | 4,53 |  |  | 3197 | 3,44 |
| Duck | Female | 1850 | 8,82 |  |  |  |  |
|  |  | 2640 | 8,69 |  |  |  |  |
|  |  | 2170 | 8,89 |  |  |  |  |
|  |  | 2300 | 8,92 |  |  |  |  |
|  |  | 2000 | 8,64 |  |  |  |  |
|  |  | 2100 | 8,70 |  |  |  |  |
|  | Male | 2700 | 7,77 |  |  |  |  |
|  |  | 2500 | 7,65 |  |  |  |  |
|  |  | 3100 | 7,79 |  |  |  |  |
|  |  | 2800 | 7,68 |  |  |  |  |
|  |  | 2600 | 7,95 |  |  |  |  |
|  |  | 2350 | 7,71 |  |  |  |  |

**S2 Table**. Final body weight and total feed intake at the time of sacrifice of the experimental birds.

Reviewer #2: Thank you for your detailed responses to the various questions raised.
The only matter you may wish to consider (and it is minor) is the use of DETOXICATION or DETOXIFICATION. The latter term is by far the most common term (a pubmed or a google search has around 10X as many entries for DETOXIFICATION compared with DETOXICATION).

**Reply**: We want to also thank again reviewer #2 for his suggestion. The word “detoxication” has been replaced by “detoxification” were appropriate.
